# Myocardial recovery in children supported with a durable ventricular assist device—a systematic review

**DOI:** 10.1093/ejcts/ezad263

**Published:** 2023-07-27

**Authors:** Sofie Rohde, Theo M M H de By, Ad J J C Bogers, Martin Schweiger

**Affiliations:** Department of Cardio-thoracic surgery, Erasmus University Medical Center, Rotterdam, Netherlands; EUROMACS, EACTS House, Windsor, United Kingdom; Department of Cardio-thoracic surgery, Erasmus University Medical Center, Rotterdam, Netherlands; Children′s Hospital Zurich, Pediatric Heart Centre, Department for Congenital Heart Surgery, Zurich, Switzerland

**Keywords:** ventricular assist device, paediatric, VAD explantation, Recovery, weaning

## Abstract

**OBJECTIVES:**

A small percentage of paediatric patients supported with a ventricular assist device (VAD) can have their device explanted following myocardial recovery. The goal of this systematic review is to summarize the current literature on the clinical course in these children after weaning.

**METHODS:**

A systematic literature search was performed on 27 May 2022 using Embase, Medline ALL, Web of Science Core Collection, Cochrane Central Register of Controlled Trials and Google Scholar to include all literature on paediatric patients supported by a durable VAD during the last decade. Overlapping study cohorts and registry-based studies were filtered out.

**RESULTS:**

Thirty-seven articles were included. Eighteen of them reported on the incidence of recovery in cohort studies, with an overall incidence rate of 8.7% (81/928). Twenty-two of the included articles reported on clinical outcomes after VAD explantation (83 patients). The aetiologies varied widely and were not limited to diseases with a natural transient course like myocarditis. Most of the patients in the included studies (70; 84.3%) were supported by a Berlin Heart EXCOR, and in 66.3% (55/83), only the left ventricle had to be supported. The longest follow-up period was 19.1 years, and multiple studies reported on long-term myocardial recovery. Fewer than half of the reported deaths had a cardiac cause.

**CONCLUSIONS:**

Myocardial recovery during VAD support is dependent on various contributing components. The interactions among patient-, device-, time- and hospital-related factors are complex and not yet fully understood. Long-term recovery after VAD support is achievable, even after a long duration of VAD support, and even in patients with aetiologies different from myocarditis or post-cardiotomy heart failure. More research is needed on this favourable outcome after VAD support.

**Figure:**
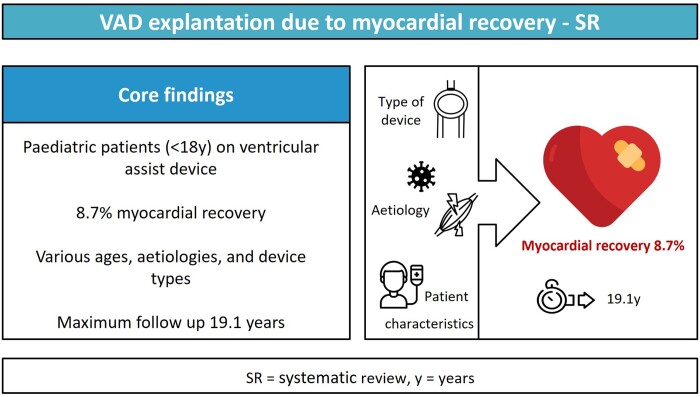

## INTRODUCTION

Every year the number of paediatric patients receiving a ventricular assist device (VAD) for end-stage heart failure is increasing, with currently over one-third of the patients receiving transplants being bridged with one [1, 2]. According to registry data, most children are bridged to a transplant, but a small number of children (7.5–11.3%) can have the VAD explanted because they undergo myocardial recovery [1, 3]. This outcome is particularly interesting because it could potentially postpone or even prevent a heart transplant and the accompanying life-long immunosuppression, morbidity and risk of death. The scarcity of donor hearts, especially among the youngest children, and the increasing waiting list times make this an even more favourable outcome [4, 5].

Although explantation of durable VADs has been reported, less is known about post-explantation outcomes. It is uncertain whether this gain in myocardial function is sustainable or if these hearts cannot last without mechanical support and deterioration at a certain point is inevitable. In adults, survival without recurrence of heart failure of 88% after 2 years has been reported [6]. In children, few small cohort studies report outcomes after recovery [7, 8], and most of what is known is based on case reports and case series [9–11]. This situation hampers evidence-based decision making regarding the weaning of the device in daily practice.

The goal of this systematic review was to search the recent literature on the explantation rate of durable VADs due to myocardial recovery in children and investigate the clinical course after explantation.

## METHODS

A systematic literature search using Embase, Medline ALL, Web of Science Core Collection, Cochrane Central Register of Controlled Trials and Google Scholar was performed by staff of the medical library of the Erasmus Medical center on 27 May 2022. Google Scholar was added only to assure that no articles were missed. The complete list of search terms can be found in the supplementary material. This systematic review was performed according to the Preferred Reporting Items for Systematic Reviews and Meta-Analyses (PRISMA) guidelines.

In this systematic review, we answered 2 questions: In the first section, we investigated the incidence of recovery in paediatric patients supported by durable VADs. To this end, all cohort studies with at least 20 children supported by a durable VAD were included and tabulated.

In the second section of this paper, the follow-up after explantation of a durable VAD due to myocardial recovery was examined. To do so, all papers reporting a clinical course after weaning were included and tabulated.

Further inclusion and exclusion criteria are depicted in Table 1.

**Table 1:** Inclusion and exclusion criteria

Inclusion criteria	Exclusion criteria
Published in the last decade (from 2012–May 2022)	No outcomes reported (transplant, death, recovery)
Paediatric patients	Adults or outcomes in adults not separately reported from outcomes in children
Durable ventricular assist devices	Extracorporeal membrane oxygenation Total artificial heart Percutaneous device Short-term device No information on the exact type of device Right ventricular assist device (RVAD)*
English	No full article (in English)
Cohort studies Case series Case reports	Abstracts Research letters Editorials Systematic reviews Meta-analyses
	Database- or registry-based studies

* Unless the percentage of patients in which only the right, non-systemic ventricle was supported was less than 3%. This cut-off was chosen arbitrarily.

Patients supported by a right ventricular assist device (RVAD) only were excluded from this review because the aetiology of right ventricle failure and recovery is essentially different from the aetiology and possible recovery of the left ventricle [12]. However, to avoid excluding large cohort studies, which are important to answer our research questions, studies with less than 3% supported by an RVAD only were included.

Two researchers (SR and MS) reviewed the papers independently and created a list of papers that met the inclusion criteria. Any discrepancies between the 2 lists were resolved through discussion until a consensus was reached.

Multiple studies were found to have overlapping cohorts. In case of overlapping study cohorts, only the most recent cohort study was included.

From the included studies, the following data—if available in the full text—were extracted:

- Information on baseline characteristics: age, sex, aetiology, prior extracorporeal membrane oxygenation (ECMO) and Interagency Registry for Mechanically Assisted Circulatory Support (INTERMACS) classification;
- Type and duration of support;
- Outcome: (cause of) death, transplant, myocardial recovery, need for mechanical circulatory support and VAD reimplant and heart function.

For the outcomes after VAD support, percentages were calculated and presented.

Due the scarcity of VAD explants after myocardial recovery and the therefore often small studies, all data, including outlier data, are presented and summarized in the tables. No statistical analysis other than calculation of percentages was performed due to the heterogeneity and the small size of the patient population.

## RESULTS

**Figure 1:**
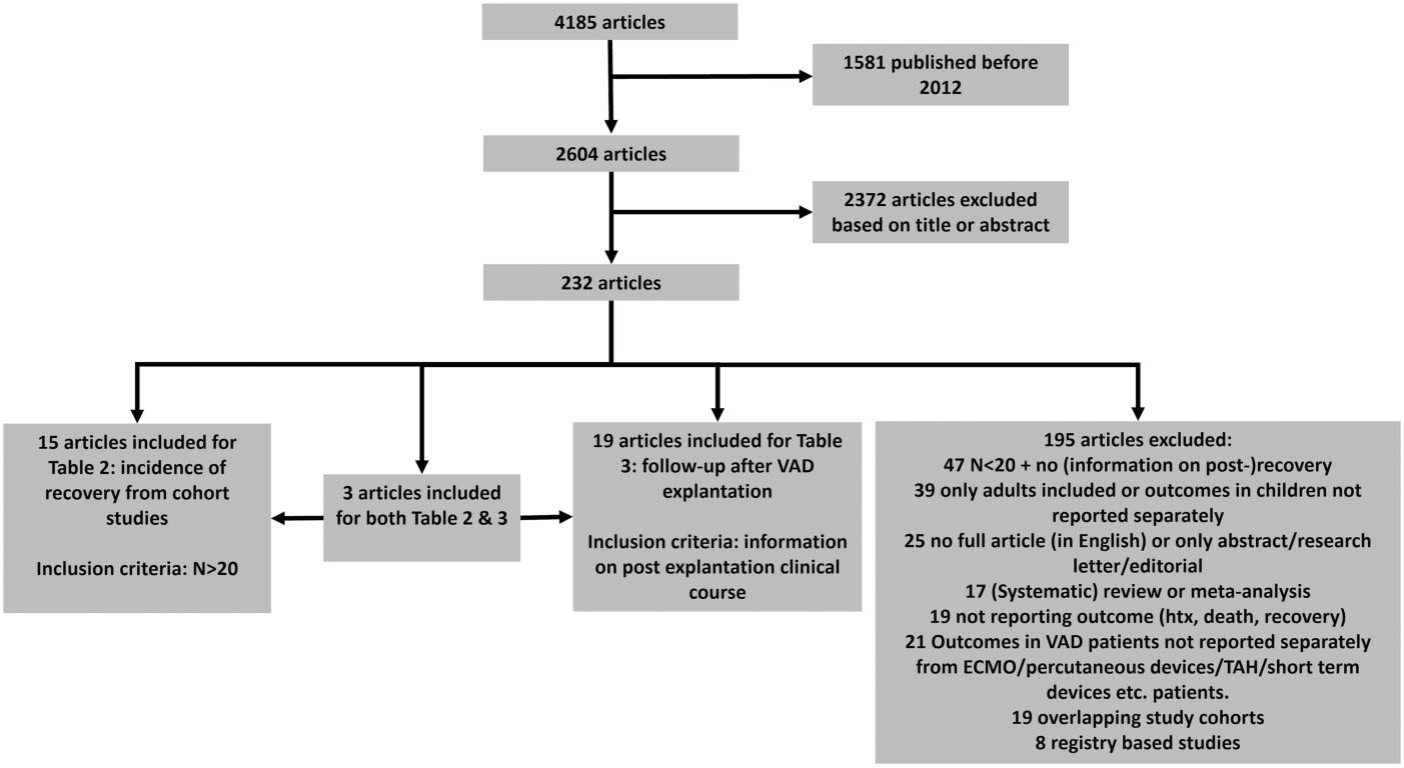
Article selection: 232/4185 articles were identified for full-text screening; 195 of the 232 articles were excluded for various reasons; 37 articles were included in the study; Table 2 was compiled from 15 articles. Nineteen articles were used to compile Table 3. Three articles were included in both tables. ECMO: extracorporeal membrane oxygenation, HTX: heart transplantation, TAH: total artificial heart, VAD: ventricular assist device.

After the search, 4185 articles were identified. A total of 1581 articles were excluded because they were published before 2012. Another 2372 articles were excluded based on title or abstract. Of the remaining 232 articles, the full text was screened and an additional 195 manuscripts were excluded. Finally, 37 articles were included in this systematic review (Fig. 1). After the first screening, the reviewers disagreed on the inclusion of 110 articles (2.6%). These disagreements were resolved through discussion.

To investigate the incidence of recovery in cohort studies (first section, Table 2), non-registry-based studies with a minimum of 20 paediatric patients supported by a durable VAD were included (18 studies). To investigate follow-up after VAD explantation (second section, Table 3), all studies that reported the clinical course in paediatric patients after the explantation of a durable VAD were included (22 studies). Three studies were included for both research questions.

**Table 2:** Incidence of recovery

	First author	Year published	Study type	Study period	Patients	Aetiology*	Prior ECMO	INTERMACS classification*	Type of device	LVAD/BiVAD	Length of support	Outcome	Recovery rate
**Europe**	Sandica [45]	2016	Single-centre cohort study	2008–2014	n = 38 61% Male	29% Myocarditis 63% CMP 8% CHD	n = 1		29 BHE 9 HVAD	30 LVAD 8 BiVAD	BHE: Med 65 days (IQR 4–619) HVAD: Med 180 days (IQR 1–1124)	27 HTx 4 Died 6 Recovered 1 Ongoing	15.8%
	Miera [7]	2018	Single-centre cohort study	1990–2016	n = 149 54% Male Med 5.8 years Med 17.0 kg	9% Myocarditis 66% CMP 25% CHD		67% 1 29% 2 5% 3 0% 4	128 BHE 32 HVAD	96 LVAD 52 BiVAD 1 RVAD	Med 37 days (IQR 10–79)	79 HTx 48 Died 21 Recovered 1 Ongoing	14.1%
	Iacobelli [17]	2017	Single-centre cohort study	2007–2016	n = 27 55% Male Med 11 months Med 6.3 kg	100% CMP			All BHE	27 LVAD	Med 147 days (IQR 86–210)	20 HTx 6 Died 1 Recovered	3.7%
	Ponzoni [14]	2021	Single-centre cohort study	2004–2021	n = 20	15% Myocarditis 45% CMP 35% CHD 5% Other/undefined			13 BHE 7 HVAD			16 HTx 4 Died	0.0%
	Pawlak [46]	2018	Multicentre cohort study	2009–2015	n = 27 61% Male Med 3.5 years Med 12.5 kg	19% Myocarditis 52% CMP 26% CHD 4% Other/undefined	n = 2	11% 1 56% 2 26% 3 4% 4 4% 5	All BHE	21 LVAD 6 BiVAD	Med 89 days (Range 6–1215)	10 HTx 8 Died 6 Recovered 3 Ongoing	22.2%
	Redondo [18]	2019	Single-centre cohort study	“the experience so far”	n = 116	“Mostly DCM”	n = 42		98 BHE 18 HVAD	83 LVAD 33 BiVAD	BHE: Mean 95 days (SD 148) HVAD: Mean 248 days (SD 346)	92 HTx 13 Died 6 Recovered 5 Ongoing	5.2%
	Rohde [19]	2020	Single-centre cohort study	2007–2018	n = 28 43% Male Med 10.7 years Med 29.9 kg	4% Myocarditis 79% CMP 18% Other/ undefined	n = 11	100% 2	All BHE	25 LVAD 3 BiVAD	Med 37 days (IQR 12–123)	14 HTx 11 Died 2 Recovered 1 Ongoing	7.1%
	Bartfay [13]	2021	Single-centre cohort study	2008–2018	n = 21 52% Male Med 5 years Mean 16 kg	10% Myocarditis 43% CMP 29% CHD 19% Other/undefined	n = 7	60% 1 40% 2–5	All BHE	15 LVAD 5 BiVAD 1 RVAD**	Med 79 days (IQR 39–239)	12 HTx 1 Died 8 Recovered	38.1%
	Fouilloux [22]	2021	Multicentre cohort study	2005–2017	n = 54 54% Male Med 17 months Med 9.8 kg	18% Myocarditis 72% CMP 6% CHD 4% Other/undefined	n = 14		All BHE	27 LVAD 27 BiVAD	Mean 623 days (Range 5–267)	32 HTx 15 Died 7 Recovered	13.0%
**North America**	Byrnes [21]	2013	Single-centre cohort study	2005–2012	n = 39 Mean 5.5 years BSA mean 0.8m^2^	44% Myocarditis 28% CMP 23% CHD 5% Other/undefined	n = 16		All BHE	21 LVAD 18 BiVAD	Mean 39 days (SD 41)	34 HTx 3 Died 2 Recovered	5.1%
	Bleiweis [20]	2022	Single-centre cohort study	2006–2022	n = 82 Med 191 days Med 5.8 kg	4% Myocarditis 39% CMP 54% CHD 4% Other/undefined			All BHE	5 LVAD 43 BiVAD 34 SVAD	Non-ongoing patients: Med 108 days (Range 4–554)	57 HTx 18 Died 5 Recovered 2 Ongoing	2.4%
	Miller [23]	2015	Single-centre cohort study	2005–2014	n = 48			All 1 or 2	40 BHE 6 HVAD 2 HMII	17 LVAD 31 BiVAD		40 HTx 7 Died/ongoing 1 Recovered	2.1%
	Stein [16]	2016	Single-centre cohort study	2004–2013	n = 50 58% Male Mean 5.4 years Mean 20.5 kg	6% Myocarditis 74% CMP 18% CHD 2% Other/undefined	n = 18		27 BHE 14 PVAD 9 HVAD or HMII	35 LVAD 15 BiVAD	Mean 75 days (range 2–906)	37 HTx 8 Died 5 Ongoing/undefined	0.0%
	Fraser [24]	2019	Single-centre cohort study	1996–2017	n = 117 51% Male	2% Myocarditis 68% CMP 21% CHD 10% Other/ undefined	n = 11	13% 1 56% 2 26% 3 2% 4 1% Unknown	48 BHE 38 HVAD 17 HMII 11 PVAD 3 DeBakey	105 LVAD 9 BiVAD 3 RVAD	Range 2–2259 days	76 HTx 19 Died 7 Recovered 15 Ongoing/undefined	6.0%
**Asia**	Sen [15]	2020	Single-centre cohort study	2009–2018	n = 27 63% Male Med 12.2 y	96.3% CMP 3.7% CHD			9 BHE 18 HVAD	27 LVAD	Med 413 days (range 30–2010)	15 HTx 5 Died 7 Ongoing/undefined	0.0%
	Sert [25]	2020	Single-centre cohort study	2014–2018	n = 21 52% Male Mean 11.0 years Mean 33.1 kg	“mostly CMP”	n = 2	Mean 2.3 ± 0.75	19 HVAD 1 HM II 1 HM 3	21 LVAD	Mean 421 days (Range 18–1460)	9 HTx 7 Died 1 Recovered 4 Ongoing/undefined	4.8%
	Komori [35]	2022	Single-centre cohort study	2015–2021	n = 20 30% Male Med 10.8 months Med 6.3 kg	15% Myocarditis 75% CMP 10% CHD	n = 10		All BHE	18 LVAD 2 BiVAD	Med 365 days (Range 241–636)	7 HTx 1 Died 5 Recovered 7 Ongoing/undefined	25.0%
**AU**	Huang [47]	2019	Single-centre cohort study	2009–2017	n = 44 52% Male	14% Myocarditis 89% CMP 18% CHD***	n = 21		34 BHE 10 HVAD	35 LVAD 5 BiVAD 4 SVAD	Med 78 days (IQR 36–144)	27 HTx 8 Died 3 Recovered 6 Ongoing/undefined	6.8%
	18 articles included, 928 patients Recovery rates between 0 and 38.8%; overall recovery rate 8.7%												

* The sum of percentages might deviate slightly from 100% due to rounded numbers.

** An RVAD supported the systemic circulation.

*** In this paper, a cardiac diagnosis was not mutually excludible, and each patient could have multiple diagnoses.

BHE: Berlin Heart EXCOR; BiVAD: biventricular assist device; CHD: congenital heart disease; CMP: cardiomyopathy; DCM: dilated cardiomyopathy; ECMO: extracorporeal membrane oxygenation; HM: HeartMate; HTx: heart transplant; HVAD: HeartWare; INTERMACS: Interagency Registry for Mechanically Assisted Circulatory Support; IQR: interquartile range; LVAD: left ventricular assist device; Med: median; PVAD: Thoratec paracorporeal ventricular assist device; RVAD: right ventricular assist device; SD: standard deviation; SVAD: single ventricular assist device.

**Table 3:** Follow-up after explantation of a ventricular assist device

	First Author	Year	Study type	Patient(s)	Aetiology	Prior ECMO	LVAD/BiVAD	Length of VAD support	Outcome after explantation	Length of Follow-up
**Berlin Heart EXCOR**	George [48]	2013	Case report	n = 1 22 Month-old-Male	PVB19 Myocarditis	Yes	LVAD	152 Days	Minimal symptoms of heart failure (NYHA I), LVEF 65%	3.5 Years
	Irving [8]	2014	Case series	n = 10 40% Male Med 1.1 years Med 10.5 kg	3 Myocarditis 4 DCM 1 HCM 1 CHD 1 Post HTx		5 LVAD 5 BiVAD	Med 36 days (Range 11–120)	6 Stable/normal cardiac function 3 VAD reimplants (8 days, 10 months and 13 months post-explant) 1 died 5 days post-explant (massive cerebral haemorrhage)	
	Sandica [45]	2016	Cohort study	n = 6			6 LVAD	Med 40.5 days (IQR 5–102)	100% Survival	Med 25.7 months (Range: 9–48)
	Urbanska [49]	2016	Case report	n = 2 3 and 3.5 Months 4.3 and 5.9 kg	2 CHD: Bland-White-Garland syndrome 3 weeks and 2 days post-operatively	No	2 LVAD	174 Days and 26 days	Both had normal circulation	
	Kohli (11]	2018	Case report	n = 1 20-Month-old female 13 kg	Pre-excitation- induced ventricular dysfunction	No	BiVAD	48 Days	Normal cardiac function	3 Years
	Fang [50]	2018	Case report	n = 1 6-Month-old male 7.4 kg	Permanent junctional reciprocating tachycardia with DCM	Yes	LVAD	47 Days	2 Weeks post-explant, EF 43%; discharged at 3 weeks	3 Weeks
	Tominaga [34]	2020	Case series	n = 4 0.4–2.1 years 6.1–8.4 kg	All DCM		4 LVAD	3.7–14 Months	Discharged home 5.6–8.2 months after explantation All doing well with no rehospitalization for heart failure	Med 24 months (IQR 17–27)
	Philip [32]	2021	Case report	n = 2 6 and 9 Months 7 and 8 kg	1 DCM 1 CHD	No	2 BiVAD	2 Months and 6 months	Both have normal cardiac function.	18 and 21 Months
	Wilkinson [51]	2021	Case report	n = 1 17-Month-old female 8.4 kg	Left main coronary artery atresia with ischaemic CMP	Yes	LVAD	59 Days	LVEF 60%	12 Months
	Delmo [30]	2021	Multi-institutional cohort study	n = 25 40% Male Med 3.4 years	11 Myocarditis 7 DCM 2 Other CMP 3 Ischaemic 1 Post-HTx 1 Post- cardiotomy	n = 2	18 LVAD 6 BiVAD 1 RVAD	Med 42 days (Range 7–700)	21 (84.0%) Alive with their native hearts 4 Died. No VAD re-implants	Med 7.8 years (range 1.3–19.1)
	Bartfay [13]	2021	Cohort study	n = 8	2 Myocarditis 1 DCM 3 CHD 2 Other/undefined		7 LVAD 1 BiVAD	Range 31–110 days	7 Survivors without recurrent heart failure 1 Died of cardiogenic shock after 3 months	
	Fouilloux [22]	2021	Cohort study	n = 7			Unknown		3 Died after 15 days (cardiac failure), 68 days (septic shock) and 109 days (ventricular arrhythmias) 4 Survived > 120 days	Range 15–120 days
	Torpocco Rivera [38]	2022	Case report	n = 2 13 Months and 3 months	1 Myocarditis 1 Multiform atrial tachycardia	n = 1	2 LVAD	3 Months and 142 days	1 Normal ventricular function 1 Discharged at 2 weeks with oral heart failure regime	18 M onths and at least 2 weeks
**HVAD**	Cavigelli-Brunner [37]	2014	Case report	n = 1 8-Year-old female BSA 0.97 m^2^	Anthracycline-induced CMP	No	LVAD	149 Days	Globally reduced biventricular function (LVEF 45%)	4 Months
	Kirk [52]	2016	Case report	n = 1 3-Year-old male 13.5 kg	Post-myocarditis DCM	No	LVAD	149 Days	Fully active, no limitations (Device in situ, decommissioned)	12 Months
	Arslan [53]	2019	Case series	n = 1 12-Year-old male	Tachycardia-induced CMP	Yes	LVAD	1 Month	After 2 years, need for a permanent pacemaker due to an intermittent complete AV block	2 Years
	Puri [33]	2022	Case series	n = 2 14 Years and 7 years	Post-chemotherapy CMP		2 LVAD	12 Months and 9 months	1 With normal ejection fraction 1 With moderately depressed biventricular systolic function (LVEF 36–46%)	6 Years and 3.5 years
**HM**	Geoffrion [10]	2021	Case report	n = 1 13-Year-old male	Myocarditis	No	LVAD	1 Day	ECMO and relisting 1 day after explant, survived to HTx	
**PVAD**	Akil [54]	2016	Case series	n = 1 15-Year-old male	Acute transplant rejection		LVAD	2 Months	Died (cause not reported)	87 Months
	Boston [9]	2019	Case report	n = 1 7 Months	CHD: DORV, hypoplastic aortic arch, common AV canal, hypoplastic RV 2-Month post stage II palliation	No	SVAD	7 Days	Alive and free from transplant and VAD re-implant	16 Months
	Merritt [55]	2022	Case series	n = 4 3.3–4.0 kg	2 SV, prior to surgical palliation 2 SV, post Norwood/hybrid	n = 4	4 SVAD	60–99 Days	2 Alive at discharge 2 Died at discharge (1 failure to wean from ventilator + mitochondrial disorder (diagnosis after explant); 1 persistent cardiopulmonary failure + concomitant MOF)	
**Jarvik**	Healy [31]	2015	Case report	n = 1 16-Year-old male	Cardiogenic shock; no aetiology was definitely diagnosed	No	LVAD*	12 Months	NYHA 1	3 Months
	22 articles included: 12 case reports, 6 case series and 4 cohort studies 83 patients, 84.3% supported by a BHE Max follow-up, 19.1 year; 14.5% died									

* Additional CentriMag RVAD for 3 days.

AV: atrioventricular; BHE: Berlin Heart EXCOR; BiVAD: biventricular assist device; BSA: body surface area; CHD: congenital heart disease; CMP: cardiomyopathy; DCM: dilated cardiomyopathy; DORV: double outlet right ventricle; ECMO: extracorporeal membrane oxygenation; HCM: hypertrophic cardiomyopathy; HM: HeartMate; HTx: heart transplantation; HVAD: HeartWare; IQR: interquartile range; LVAD: left ventricular assist device; LVEF: left ventricular ejection fraction; Med: median; MOF: multiple organ failure; NYHA: New York Heart Association; PVAD: Thoratec paracorporeal ventricular assist device; PVB19: parvovirus B19; RV: right ventricle; RVAD: right ventricular assist device; SV: single ventricle; SVAD: single ventricular assist device.

### Incidence of recovery

To investigate the incidence of recovery in the cohort studies, 18 papers were included and sorted by continent (Table 2). Recovery rates between 0 and 38.8% were reported; however, only 1 study reported an incidence rate above 25% [13], and most of the studies were fairly small. In this study, children were supported by a Berlin Heart EXCOR (BHE) (Berlin Heart GmbH, Berlin, Germany) and had a median age of 5 years. In 23.8% (5/21) of the patients, a biventricular assist device (BiVAD) was used. The mortality rates in this study were remarkably low (1/21; 4.8%), even though 60% of the patients were classified as INTERMACS I and one-third (7/21) were supported by an ECMO prior to VAD support [13]. Furthermore, 3 studies [14–16] reported an incidence of recovery of 0.0%, and 12 of the 18 studies included showed an incidence of recovery lower than 10% [14–25]. The studies including more than 100 patients reported an incidence of recovery of 5.2–14.1% [7, 18, 24]. Overall, 81 of the 928 patients included in this systematic review had their VAD explanted due to myocardial recovery (8.7%).

### Follow-up after recovery

Twenty-two articles were included to investigate outcomes after the durable VADs were explanted. Most of these articles were case reports (12/22) or case series (6/22). Table 3 presents the 22 included studies sorted by type of device. In most of these studies, the durable VAD used was the BHE (13 of the 22 included studies), followed by the HeartWare (Medtronic, Framingham, MA, USA) (HVAD; 4 of the 22 included studies).

**Figure 2:**
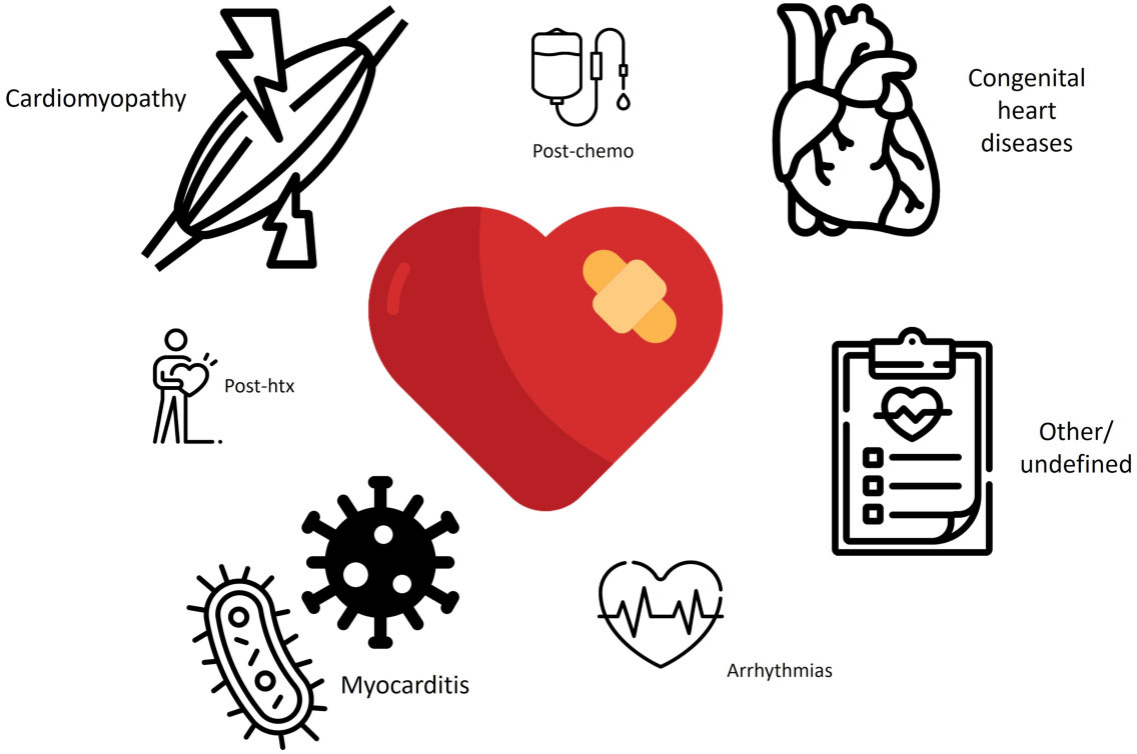
Range of aetiologies in patients with myocardial recovery after VAD support. Aetiologies are scaled based on the prevalence of diagnosis reported by the studies included in table 3. Of note, many studies reported overlapping/concomitant aetiologies, and this is not meant as a representation of the actual incidence of the different types of aetiology in this subpopulation. This figure has been designed using images from Flaticon.com. Tx: heart transplantation

In the 22 identified articles, a total of 83 patients with durable devices were identified. Aetiology varied widely and was not limited to diseases with a natural transient course like myocarditis (Fig. 2). Structural heart diseases (other than in the case of post-cardiotomy heart failure) and even a few cases of ischaemic cardiomyopathy were included. Most of the patients (70; 84.3%) were supported by a BHE followed by the paracorporeal Thoratec Ventricular Assist Device (PVAD; 6; 7.2%) (Thoratec, Pleasanton, CA, USA) and an HVAD (5; 6.0%). In 55 (66.3%) patients, a left ventricular assist device (LVAD) was implanted; in 15 (18.1%), a BiVAD; in 1 (1.2%) an RVAD; and in 5 (6.0%), a single ventricular assist device. One study (with 7 patients included) did not report on the type of support.

The duration of follow-up varied widely, and the maximum follow-up was 19.1 years. Twelve of the 83 patients (14.5%) died during the follow-up period. Causes of death varied and were not all related to heart failure. Cardiac causes of death included cardiogenic shock in 1 patient, cardiac failure in 1, ventricular arrhythmias in 1 and persistent cardiopulmonary failure (hypoxia and hypercarbia) with concomitant multiple organ failure in 1. Other causes of death included a massive haemorrhagic cerebrovascular accident in 1, septic shock in 1 and redirection of care due to mitochondrial disease plus failure to wean from ventilator support and poor long-term prognosis in 1. In 5 cases the cause of death was not reported (Tables 3, 4).

**Table 4:** Causes of death after ventricular assist device explantation due to myocardial recovery

Cardiac causes (n)		Other causes (n)	
Cardiogenic shock	1	Haemorrhagic cerebrovascular accident	1
Cardiac failure	1	Bronchiolitis	1
Ventricular arrhythmias	1	Septic shock	1
Persistent cardiopulmonary failure (hypoxia and hypercarbia) with concomitant multiple organ failure	1	Redirection of care due to mitochondrial disease plus failure to wean from ventilator support and poor long-term prognosis	1
		Sudden death	2
		Unknown	5

During the follow-up period, at least 3 patients needed VAD reimplants and 2 needed cardiac transplants. Caution should be taken since the different studies reported outcomes in various ways. For example, some studies only reported a survival rate, without describing cardiac function.

## DISCUSSION

This systematic review summarizes the recent literature on the incidence of myocardial recovery in children supported with a durable VAD. Most studies reported an incidence between 0 and 15% with an overall incidence of 8.7%. This finding is in accordance with the reported incidence of recovery in large registries [1, 3].

The researchers reported a wide range of recovery rates up to 38.1% [13]. Incidence rates can be influenced by various factors. First, aetiology: A population with a higher percentage of more transient types of diseases, e.g. acute myocarditis or post-cardiotomy failure, is more likely to have higher incidence rates of recovery compared to progressive or structural diseases. After bridging the acute phase with VAD support, the natural course of the disease allows improvement in cardiac function and weaning from the device. Myocarditis was associated with higher myocardial recovery [odds ratio (OR) 17.56, 95% confidence interval (CI) 4.6–67.4) in a study from Miera et al. that included almost 150 patients [7]. However, our group performed a multivariable Cox regression analysis using the paediatric European Registry for Patients with Mechanical Circulatory Support (EUROMACS) on 303 BHE-supported children and found no significant association between primary diagnoses and recovery (non-CHD versus CHD: hazard ratio 0.919, 95% CI 0.525–1.611, *P* = 0.771) [26]. This review also showed that myocarditis and post-cardiotomy failure are not the only diagnoses associated with successful VAD explantation due to recovery.

Second, the type of device seems to matter. It is thought that the key mechanism to myocardial recovery is left ventricular unloading, which pulsatile devices seem to achieve better than continuous devices [27]. In a study that included close to 400 patients (paediatric and adult), patients with pulsatile-flow LVAD were almost 3 times more likely to have their device explanted due to a gain in myocardial function [28]. The majority of patients included in this systematic review were supported by pulsatile devices. This fact might support the aforementioned study, although no strong conclusions can be drawn from it.

Third, the duration of VAD support is likely to affect recovery. A diseased heart needs sufficient time to recover but, on the other hand, it is plausible that in children in whom recovery does not occur in the first months, myocardial recovery is unlikely to happen suddenly thereafter. This observation is supported by the results of a molecular study by Madigan et al. [29] that show maximum structural reverse remodelling by about 40 days. However, multiple studies included in this systematic review report on cases of myocardial recovery after 6 months of support or more [30–34]. Moreover, a potential late recovery might be missed if a donor heart becomes available early before recovery occurs.

Another factor that is sometimes suggested to influence recovery rates is the general condition of the patient. One could argue that sicker children (reflected for example in their INTERMACS classification or the need for ECMO prior to VAD support) have less favourable outcomes and therefore a lower chance of myocardial recovery. On the other hand, in the included study with the highest recovery rate (38.1%), 60% of the children were classified as INTERMACS I and one-third were supported by ECMO prior to VAD support [13]. INTERMACS classification, previous ECMO or previous intubation was not significantly associated with multivariable analyses in BHE-supported children [26].

Furthermore, age and body surface area are associated with myocardial recovery. A previous report from our group showed significantly higher recovery rates in BHE-supported children with a body surface area < 0.53 m^2^ compared to children with a higher body surface area (21.8% vs 4.3–7.6% at 1 year, *P* = 0.00534) [26]. Similarly, Miera et al. [7] showed a more than fivefold increased recovery rate for children under 2 years of age (OR 5.64, 95% CI 2.0–16.6). Some of our included studies seem to reflect this too. For example, some studies with older patients show lower recovery rates [15, 16, 21, 25], whereas others with younger children show higher recovery rates [22, 35].

Lastly, the lack of universally accepted, evidence-based protocols for both implanting and possible weaning of VAD support are likely to shape outcome as well as recovery rates [36]. In the last few years, a few protocols have been suggested in the literature [7, 8, 32, 34, 37, 38]. Although most of them use echocardiographic parameters and vital signs such as heart rate and blood pressure to evaluate myocardial recovery, substantial differences exist among them. For example, invasive haemodynamic measurements are often done, but various thresholds are used [7, 34, 37, 38], and some centres are not convinced of the added value of Swan Ganz measurements to select patients for VAD explantation [8]. Additionally, a minimum duration of support is sometimes included in the criteria before myocardial recovery is evaluated [8, 34], and the duration of off-pump trials varies widely [7, 32, 34, 38]. Furthermore, not all patients who are weaned meet all weaning criteria [37]. Suboptimal parameters can be accepted in a specific patient with high complication rates. This so-called “forced” weaning could potentially be less successful. Additionally, some institutional protocols use a temporary mechanical circulatory support system instead of a durable one if recovery can be expected.

One important question after durable VAD explantation remains long-term follow-up. As seen in this review, many studies do not report long-term data, but some papers show that long-term myocardial recovery after a VAD is explanted is achievable. Twelve of the 83 (14.5%) patients previously supported by a durable VAD died during follow-up. The actual proportion might be higher due to limited follow-up time in some papers and due to publication bias. Post-explantation causes of death were not always cardiac, although most of them probably are related to the hospitalization and a certain general weakness or vulnerability of the body linked to the underlying original cardiac diagnosis. Furthermore, at least 5 children had to undergo ECMO support, a VAD reimplant or a heart transplant (HTx). Besides the aforementioned reasons for possibly underestimated mortality rates, which also apply to the rates of ECMO/VAD and/or HTx, the latter might also be higher due to the various ways of reporting on outcomes in the different case reports. For example, sometimes only a survival percentage is mentioned, without further specifying any possible recurrent heart failure.

In adults, explantation due to myocardial recovery only occurs in a small percentage of the patients [39, 40]. Independent predictors for this outcome were—inter alia—younger age (<50 year; OR 2.5), non-ischaemic aetiology (OR 5.4%), time since initial diagnosis (<2 years; OR 3.4) and type of device (axial vs centrifugal flow; OR 7.6) [41]. Remarkably, only a small percentage of patients with substantial recovery undergo explantation [41, 42]. Suggested reasons for this discrepancy are uncertainty regarding post-explant outcomes, limited experience with explanting VADs and a heart transplant being the “gold standard” of treatment [43, 44].

## LIMITATIONS

This systematic review summarizes the available literature on the incidence and follow-up after weaning from a durable VAD due to myocardial recovery. Conclusions from this study have to be interpreted cautiously because of the high heterogeneity of this population. Children of all ages, with various aetiologies and INTERMACS classifications, supported for different durations by different types of VADs, were included. A meta-analysis was not possible due to the heterogeneity of the population; thus this paper merely serves as an overview of what is known. No analyses to determine risk factors for recovery could be done.

Another important limitation of this study is publication bias. It is possible that, especially for case reports on follow-up after recovery, successful clinical courses were more likely to be reported and published. Furthermore, this review depended on what was reported. Therefore, we were limited to providing a homogeneous and extensively detailed overview. For example, it cannot be ruled out that some of the devices registered as LVAD or RVAD were actually single ventricular assist devices.

Furthermore, although pharmacological therapy is of paramount importance in this specific population, drug therapy was rarely reported in the included studies. Therefore, it was not mentioned further in this manuscript.

Lastly, one must consider that the VAD experience summarized here is an experience from the last few decades. In these years, many potentially contributing factors have changed: Experience has increased, devices have evolved and protocols have been developed and altered. This situation hampers the interpretation of the results.

## CONCLUSION

Myocardial recovery during VAD support is a scarce but the most favourable outcome, especially in light of high waiting list mortality, scarcity of donor hearts and the higher morbidity and mortality after a cardiac transplant. This outcome is, however, dependent on various contributing components. The interactions among patient- (age, aetiology, INTERMACS classification), device- (pulsatile/continuous, LVAD/BiVAD), time- (duration of support, duration of disease, duration of follow-up) and hospital- (experience, protocols used) related factors are complex and not yet fully understood. However, long-term survival after explanting a VAD seems achievable, even after long-term VAD support and even in patients with aetiologies different from myocarditis or post-cardiotomy heart failure. This valuable outcome should be better investigated in order to optimize this therapeutic strategy. To do so, joint efforts are needed, and we encourage large registries to include follow-up data after explantation.

## Supplementary Material

ezad263_Supplementary_Data

## Data Availability

The data underlying this article are available in the article and in its online supplementary material.

## REGISTRATION

This systematic review was not officially registered prior to performing it.
