# Supplementary material for: Myocardial recovery in children supported with a durable ventricular assist device—a systematic review
Source: Eur J Cardiothorac Surg. 2023 Jul 27;64(2):ezad263. doi: 10.1093/ejcts/ezad263 (PMC10560320; doi:10.1093/ejcts/ezad263)
Supplement: ezad263_Supplementary_Data [file ezad263_supplementary_data.docx]

**Supplementary material**

**Search term**

**embase.com**

('ventricular assist device'/exp OR 'heart assist device'/de OR (((ventric* OR biventric* OR heart* OR cardiac OR circulat*) NEAR/3 assist* NEAR/3 (device* OR unit OR units)) OR ((vad OR vads) NOT ((vascul* NEAR/3 access*) OR vitamin-a)) OR lvad OR rvad OR hvad OR bivad OR lvads OR rvads OR hvads OR bivads OR (berlin NEXT/1 (heart OR incor OR excor)) OR heartware OR Heartmate* OR DeBakey OR Jarvik OR HeartAssist-5 OR Thoraretec OR PVAD OR Levitronic* OR abiomed OR marquet-rotaflow* OR sorin-revolution* OR Tandem-heart*):ab,ti,kw) AND (child/exp OR adolescent/exp OR adolescence/exp OR 'child behavior'/de OR 'child parent relation'/de OR pediatrics/exp OR childhood/exp OR 'child nutrition'/de OR 'infant nutrition'/exp OR 'child welfare'/de OR 'child abuse'/de OR 'child advocacy'/de OR 'child development'/de OR 'child growth'/de OR 'child health'/de OR 'child health care'/exp OR 'child care'/exp OR 'childhood disease'/exp OR 'child death'/de OR 'child psychiatry'/de OR 'child psychology'/de OR 'pediatric ward'/de OR 'pediatric hospital'/de OR 'pediatric anesthesia'/de OR 'pediatric intensive care unit'/de OR 'neonatal intensive care unit'/de OR (adolescen* OR preadolescen* OR infan* OR newborn* OR (new NEXT/1 born*) OR baby OR babies OR neonat* OR child* OR kid OR kids OR toddler* OR teen* OR boy* OR girl* OR minors OR underag* OR (under NEXT/1 (age* OR aging)) OR juvenil* OR youth* OR kindergar* OR puber* OR pubescen* OR prepubescen* OR prepubert* OR pediatric* OR paediatric* OR school* OR preschool* OR highschool*):ab,ti,kw) NOT ([animals]/lim NOT [humans]/lim) NOT (([Conference Abstract]/lim AND [1800-2020]/py) OR [Letter]/lim OR [Note]/lim OR [Editorial]/lim) AND [english]/lim

**Medline Ovid**

(Heart-Assist Devices/ OR (((ventric* OR biventric* OR heart* OR cardiac OR circulat*) ADJ3 assist* ADJ3 (device* OR unit OR units)) OR ((vad OR vads) NOT ((vascul* ADJ3 access*) OR vitamin-a)) OR lvad OR rvad OR hvad OR bivad OR lvads OR rvads OR hvads OR bivads OR (berlin ADJ (heart OR incor OR excor)) OR heartware OR Heartmate* OR DeBakey OR Jarvik OR HeartAssist-5 OR Thoraretec OR PVAD OR Levitronic* OR abiomed OR marquet-rotaflow* OR sorin-revolution* OR Tandem-heart*).ab,ti,kf.) AND (exp Child/ OR exp Infant/ OR exp Adolescent/ OR exp "Child Behavior"/ OR exp "Parent Child Relations"/ OR exp "Pediatrics"/ OR "Child Nutrition Sciences"/ OR "Infant nutritional physiological phenomena"/ OR exp "Child Welfare"/ OR "Child Development"/ OR exp "Child Health Services"/ OR exp "Child Care"/ OR "Child Rearing"/ OR exp "Child development Disorders, Pervasive"/ OR "Child Psychiatry"/ OR "Child Psychology"/ OR "Hospitals, Pediatric"/ OR exp "Intensive Care Units, Pediatric"/ OR (adolescen* OR infan* OR newborn* OR (new ADJ born*) OR baby OR babies OR neonat* OR child* OR kid OR kids OR toddler* OR teen* OR boy* OR girl* OR minors OR underag* OR (under ADJ1 (age* OR aging)) OR juvenil* OR youth* OR kindergar* OR puber* OR pubescen* OR prepubescen* OR prepubert* OR pediatric* OR paediatric* OR school* OR preschool* OR highschool*).ab,ti.) NOT (exp animals/ NOT humans/) NOT (news OR comment* OR editorial* OR abstract* OR book* OR chapter* OR dissertation abstract*).pt. AND english.la.

**Cochrane CENTRAL**

((((ventric* OR biventric* OR heart* OR cardiac OR circulat*) NEAR/3 assist* NEAR/3 (device* OR unit OR units)) OR ((vad OR vads) NOT ((vascul* NEAR/3 access*) OR vitamin NEXT a)) OR lvad OR rvad OR hvad OR bivad OR lvads OR rvads OR hvads OR bivads OR (berlin NEXT/1 (heart OR incor OR excor)) OR heartware OR Heartmate* OR DeBakey OR Jarvik OR HeartAssist NEXT 5 OR Thoraretec OR PVAD OR Levitronic* OR abiomed OR marquet NEXT rotaflow* OR sorin NEXT revolution* OR Tandem NEXT heart*):ab,ti,kw) AND ((adolescen* OR preadolescen* OR infan* OR newborn* OR (new NEXT/1 born*) OR baby OR babies OR neonat* OR child* OR kid OR kids OR toddler* OR teen* OR boy* OR girl* OR minors OR underag* OR (under NEXT/1 (age* OR aging)) OR juvenil* OR youth* OR kindergar* OR puber* OR pubescen* OR prepubescen* OR prepubert* OR pediatric* OR paediatric* OR school* OR preschool* OR highschool*):ab,ti,kw) NOT "conference abstract":pt

**Web of science**

TS=((((ventric* OR biventric* OR heart* OR cardiac OR circulat*) NEAR/2 assist* NEAR/2 (device* OR unit OR units)) OR ((vad OR vads) NOT ((vascul* NEAR/2 access*) OR vitamin-a)) OR lvad OR rvad OR hvad OR bivad OR lvads OR rvads OR hvads OR bivads OR (berlin NEAR/1 (heart OR incor OR excor)) OR heartware OR Heartmate* OR DeBakey OR Jarvik OR HeartAssist-5 OR Thoraretec OR PVAD OR Levitronic* OR abiomed OR marquet-rotaflow* OR sorin-revolution* OR Tandem-heart*) AND (adolescen* OR preadolescen* OR infan* OR newborn* OR (new NEAR/1 born*) OR baby OR babies OR neonat* OR child* OR kid OR kids OR toddler* OR teen* OR boy* OR girl* OR minors OR underag* OR (under NEAR/1 (age* OR aging)) OR juvenil* OR youth* OR kindergar* OR puber* OR pubescen* OR prepubescen* OR prepubert* OR pediatric* OR paediatric* OR school* OR preschool* OR highschool*) NOT ((animal* OR rat OR rats OR mouse OR mice OR murine OR dog OR dogs OR canine OR cat OR cats OR feline OR rabbit OR cow OR cows OR bovine OR rodent* OR sheep OR ovine OR pig OR swine OR porcine OR veterinar* OR chick* OR zebrafish* OR baboon* OR nonhuman* OR primate* OR cattle* OR goose OR geese OR duck OR macaque* OR avian* OR bird* OR fish*) NOT (human* OR patient* OR women OR woman OR men OR man))) AND DT=(article OR review OR early access OR letter) AND LA=(english)

**Google scholar**

"ventricular|heart|cardiac assist device|devices"|"berlin heart"|heartware|Heartmate|DeBakey|Jarvik|"HeartAssist-5"|Thoraretec|Levitronic|abiomed|"marquet rotaflow"|"sorin revolution"|"Tandem heart" children|pediatric|paediatric|pediatrics|paediatrics
